# Supplementary material for: Usefulness of lung sound data collection using Skeeper SM-300® device: A pilot study
Source: PLoS One. 2026 May 13;21(5):e0348588. doi: 10.1371/journal.pone.0348588 (PMC13170829; doi:10.1371/journal.pone.0348588)
Supplement: Attachment — Submitted filename: Review of PONE-D-25-42773_R1.pdf [file pone.0348588.s007.pdf]

Remove detail of your intial **5-level scale of audible acceptability** (see page 9, ln 166-174) and references to this elsewhere. The **change to just three categories** (negative, equal, positive) is logical and will not only appeal to this reviewer but to all readers of your manuscript.

Shorten text on pg. 21, ln 280-291

There were ~~even~~ cases where the classification of breath sounds decided by the Coordinator differed from the opinions of the majority of the Raters, so the Coordinator listened to the digitally recorded breath sounds againand checked the Mel-Spectrogram of the breath sounds, and in some cases, the Coordinator's original judgment was revised. This could have occurred because the Coordinator did not listen to the breath sounds with a traditional stethoscope at each site for a whole minute before classifying them as one of the four breath sounds. Considering the limited time for detailed auscultation in doctor's offices and clinics, Skeeper-recorded breath sounds of patients at home are less constrained by time and thus may provide important clues to clinicians~~. In addition, breathing sounds from the Skeeper device is very similar to that of a traditional stethoscope in that it records and reproduces a wide frequency range and high-frequency breath sounds, and is superior to a stethoscope that uses a piezoelectric sensor. This will be described later.~~

Legend for Table 3

Your **explanation** for the large number of missing values for Rater 1 that you provide to me in your answer **has to be added** so that all readers who might wonder about possible reasons can understand, e.g., Rater 1 noticed more artifacts from skin friction, low lung sound level during shallower breathing, and/or loud ambient noise. The respective recordings were not counted for him.
