# Supplementary material for: Usefulness of lung sound data collection using Skeeper SM-300® device: A pilot study
Source: PLoS One. 2026 May 13;21(5):e0348588. doi: 10.1371/journal.pone.0348588 (PMC13170829; doi:10.1371/journal.pone.0348588)
Supplement: Attachment — Submitted filename: Response to Reviewers.docx [file pone.0348588.s009.docx]

Reviewer #1: Please as this is the pilot study, you should mention it in the title of the manuscript: Usefulness of Lung Sound Data Collection Using Skeeper SM-300 ® Device: A pilot study

- Thank you for your kind comment and recommendation. We modified the title as requested.

Reviewer #3: I appreciate the changes you made. There are a few remaining issues that you can also see on an uploaded PDF file where I have used strike-out and highlighting for suggested modifications.

Remove detail of your initial 5-level scale of audible acceptability (see page 9, ln 166-174) and references to this elsewhere. The change to just three categories (negative, equal, positive) is logical and will not only appeal to this reviewer but to all readers of your manuscript.

- Thank you for your kind comment and recommendation. We are terribly sorry for this mistake we made and corrected our error in our manuscript as recommended. Please refer to the revised manuscript (we made a green-colored mark on the requested modification on page 8 and 9.

Shorten text on pg. 21, ln 280-291

There were cases where the classification of breath sounds decided by the Coordinator differed from the opinions of the majority of the Raters, so the Coordinator listened to the digitally recorded breath sounds again and checked the Mel-Spectrogram of the breath sounds, and in some cases, the Coordinator's original judgment was revised. This could have occurred because the Coordinator did not listen to the breath sounds with a traditional stethoscope at each site for a whole minute before classifying them as one of the four breath sounds. Considering the limited time for detailed auscultation in doctor’s offices and clinics, Skeeper-recorded breath sounds of patients at home are less constrained by time and thus may provide important clues to clinicians.

🡪 We appreciate your meticulous editing of our manuscript and modified several sentences identical to the PDF file you have sent us. Please refer to the change with green-colored mark in our manuscript on page 21.

Legend for Table 3
Your explanation for the large number of missing values for Rater 1 that you provide to me in your answer has to be added so that all readers who might wonder about possible reasons can understand, e.g., Rater 1 noticed more artifacts from skin friction, low lung sound level during shallower breathing, and/or loud ambient noise. The respective recordings were not counted for him.

- Thank you for your comment. We added sentences in your PDF file to the legend of Table 3.
